# Keystone Perforator Island Advancement Flap as Another Option for Closure after Radical Excision of Axillary Hidradenitis Suppurativa

**DOI:** 10.1055/a-2575-1211

**Published:** 2025-07-23

**Authors:** Timea H. Virag, Geoffrey G. Hallock, Ileana R. Matei, Alexandru V. Georgescu

**Affiliations:** 1 Clinic of Plastic, Aesthetic Surgery and Reconstructive Microsurgery, University of Medicine Iuliu Hatieganu, Clinical Hospital of Rehabilitation, Cluj Napoca, Cluj, Romania; 2 Division of Plastic Surgery, Sacred Heart Campus, St. Luke's Hospital, Allentown, Pennsylvania

**Keywords:** hidradenitis suppurativa, axilla, keystone perforator island flap

## Abstract

Surgical treatment of axillary hidradenitis suppurativa (HS) requires wide excision followed by definitive wound closure to not limit shoulder function. An international prospective study was undertaken to compare how a keystone perforator island advancement flap can be a reliable solution. Independently, two Transatlantic institutions, from 2019 to 2024, surgically resected axillary HS followed by a keystone perforator flap for defect management. A total of 23 patients were treated, including 4 with bilateral disease, employing 27 total flaps that permitted the comparison of demographic data, Hurley disease stage, flap viability and complications, need for secondary surgeries, duration of treatment, and functional outcome. Demographic data in both countries was similar with regard to mean age, female preponderance, presence of obesity, and prior means of treatment. Surgical treatment was not limited in either country only to Hurley stage III individuals. In Romania, 6/11 (54.5%) compared to 3/12 (25.0%) of U.S. patients were classified as Hurley stage II. No flap necrosis was observed, allowing unrestricted shoulder mobility within 3.5 (2–4) months in Romania, but more slowly by 4.4 (3–9) months in the United States. All complications were minor, occurring in 11 (43.5%) patients, albeit three times more frequently in the United States. Overall, the most common problem was lesser curvature dehiscence (63.6%). No disease recurrences were noted. The keystone perforator island advancement flap is a safe, rapid, and simple local flap option for the closure of the axillary defect after the excision of HS. The learning curve was straightforward, so reconstructive surgeons can readily adapt to utilize this approach.

## Introduction

Hidradenitis suppurativa (HS) is an inflammatory disease of folliculopilosebaceous units most commonly found in the axillary or inguinal regions. 1 2 Consequently, some have renamed this acne inversa. 2 The etiology is unclear, possibly genetic, immunological, or gender-related, with smoking and obesity considered to be associated high-risk factors. 3 4 The high worldwide prevalence rate of up to 4% makes this an astonishingly common problem. 3 Commonly encountered are chronic draining sores that are painful and disabling. These limit mobility and undermine social as well as interpersonal relationships, often resulting in diminished work productivity and an inferior quality of life. 4 5 Milder forms of HS may be controlled using medical options or biologic therapies, but recurrence is predictable upon the cessation of any of these agents. 1 4 Surgical intervention in some form may be a more permanent treatment. To minimize the risk of axillary HS recurrence, wide excision of all hair-bearing regions into surrounding normal tissues may be the best means for cure. 3 5 6 7 Many then advocate the use of a local flap to close the resultant defect to promote early healing and, for axillary HS, more rapid restoration of full mobility. 1 2 5 7 8 9 Specifically, a keystone perforator island advancement flap in Romania 6 as well as the United States was independently selected as the local flap solution for axillary HS, as this appeared to be a simpler perforator flap alternative that is easy to harvest since there is no need to identify nor dissect any specific perforator, and multiple perforators are usually readily available to make this a consistently reliable option.

## Ideas

A prospective Transatlantic study from 2019 to 2024 included all patients with axillary HS that were treated surgically by appropriate wide excision and then defect closure with a keystone perforator island advancement flap. Both Hurley stage III and Hurley stage II patients intolerant of alternatives agreed to this approach. This encompassed 23 patients who had a total of 27 keystone flaps, as 4 had bilateral HS.

Demographic data included age, gender, presence of obesity (arbitrarily defined as body mass index [BMI] >30) or any other comorbidities, and acknowledgement of any prior treatment, whether medical or surgical ( Table 1 ). Assessment of flap viability, complications including any secondary surgical intervention, need for hospital admission, and duration until both healing and rehabilitation were adequate to restore shoulder function were also tabulated ( Table 2 ). Informed consent was obtained from all patients to appropriately allow the use of their clinical photographs or other data for this research, educational, and publication purposes. This study protocol also conformed to the ethical guidelines of the 1975 Declaration of Helsinki.

**Table 1:** Demographic data for axillary hidradenitis suppurativa treated with a keystone perforator island advancement flap

	United States	Romania	Overall
Number of patients	12 (52.2%) a	11 (47.8%) a	23
Mean age (years; range)	36.6 (24–56)	43.7 (21–72)	40.4 (21–72)
Gender			
Male	2 (16.7%) b	3 (27.3%) b	5 (21.7%) a
Female	10	8	18
Obesity c	5 (41.7%) b	3 (27.3%) b	8 (34.8%) a
Comorbidities			
Diabetes mellitus	3	1	4
Hypertension	2	1	3
Prior treatment			
Medical	–	–	–
Surgical incision and drainage	2	2	4

a Percentage of overall total.

b Percentage of subset.

c Defined as body mass index (BMI) >30.

**Table 2:** Institutional comparison of clinical evaluation and results after treatment of axillary hidradenitis suppurativa using a keystone perforator island flap

	United States	Romania	Overall
Hurley stage disease			
Stage II			
Unilateral	3	5	8
Bilateral	–	1	1
Stage III			
Unilateral	8	3	11
Bilateral	1	2	3
Total keystone flaps	13 (48.1%) a	14 (51.9%) a	27
Hospital admission			
Inpatient	–	11	11
Outpatient	12	–	12
Flap viability			
Complete	13	14	27
Partial (%) loss	0	0	0
Concomitant brachioplasty	3 (23.1%) b	–	–
Complications			
Dehiscence			
Lesser curvature	5	2	7
Greater curvature	1	–	1
Delayed healing (other)	2	–	2
Infection	1	–	1
Total complications	9 (69.2%) b	2 (14.3%) b	11 (43.7%) a
Secondary surgery	None	None	None
Functional recovery	NL	NL	NL
Mean duration of treatment (range) c	4.4 (3–9)	3.5 (2–4)	4.0 (2–9)

Abbreviation: NL, no limitation.

a Percentage of overall total.

b Percentage of subset.

c From date of surgery until rehabilitation with full shoulder mobility completed (months).

### Surgical Technique

**Video 1** “Axillary keystone flap” demonstrates the surgical steps when a keystone flap is used for the treatment of axillary hidradenitis suppurativa and presents a typical clinical result.


**Fig. 1:**
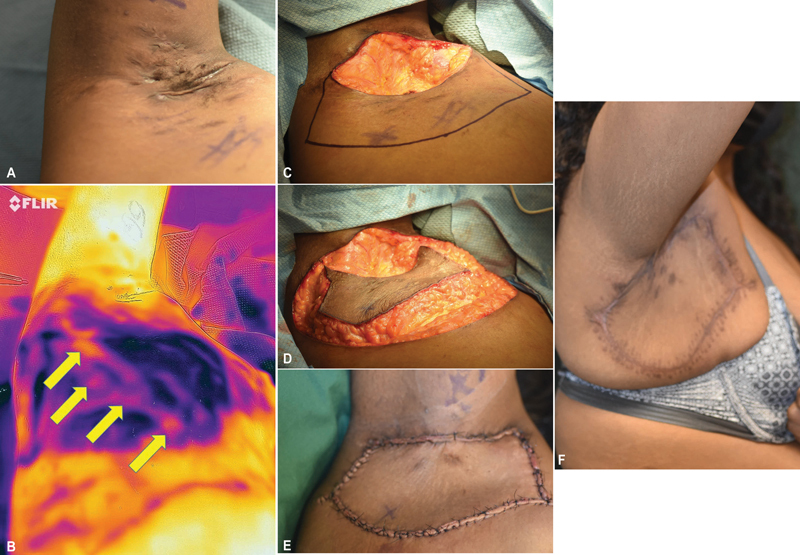
( **A** ) Long-standing Hurley stage III right axillary hidradenitis suppurativa (HS) existing since teenage years. ( **B** ) Thermal image following preoperative “cold challenge” revealed multiple “hotspots” (arrows) corresponding to perforators on lateral thorax inferior to the region of disease. ( **C** ) Following wide excision of HS, keystone flap designed on the mobile fat pad inferior to the defect, with vertical height slightly greater than that of the defect to ensure inclusion of the “hotspots” marked “x.” ( **D** ) In situ flap appearance after completion of peripheral incisions and release of superficial adipose layer. ( **E** ) Completion of flap insetting. ( **F** ) The outcome 4 months later demonstrates unrestricted upper extremity elevation and typical scar residue from a keystone perforator island advancement flap.

At both institutions, wide excision of all hair-bearing skin, nodules, abscesses, and sinus tracts in the axilla or neighboring regions, as well as removal of any suspected subcutaneous tissue reservoirs of disease, was precisely accomplished. Any extensions into the upper humeral region can normally be closed primarily as would be done in a brachioplasty. 7 A stepwise approach for closure of the axilla itself by use of an axillary keystone perforator island advancement flap is reiterated ( Video 1 : Axillary keystone flap, and Fig. 1 ) as follows. The regions inferior to the surgical defect, along the posterior trunk or anteriorly toward the breast, should be pinched to determine the degree of soft tissue flexibility that would allow facile advancement of a block of tissue comparable in size to that of the excision defect. A pair of tangents of length slightly exceeding the vertical height of the defect should be drawn from either side onto the chosen donor site. An arc connecting these tangents will form the greater curvature of the proposed keystone flap.

All marked borders are incised to first separate the superficial from the deep subcutaneous layer. If this does not allow the necessary flap advancement for insetting, the deep subcutaneous tissues or even the dorsal thoracic fascia must be incised first along its greater curvature, 10 or then sometimes also along the lateral incisions if necessary. Especially if some rotation is needed, subfascial undermining can be performed—avoiding “hotspots” corresponding to perforators 11 ( Video 1 : Axillary keystone flap)—until the desired reach is finally achieved without unreasonable tension.

A layered closure best secures insetting first along the lesser curvature, whereas only skin is coapted on the other three sides of the flap, which better reduces the final tension. Some form of surgical pillow worn at the hip reduces the risk of arm adduction crushing the flap and jeopardizing its blood supply. Sutures should not be removed until healing is sufficient, which typically is several weeks later.

Although the arm can be elevated to the horizontal almost immediately postsurgery, a full range of motion must be restricted until after completion of suture removal; otherwise, dehiscence, especially of the lesser curvature, may be a sequela.

### Results

Both the United States and Romania groups with axillary HS were similar in number; the majority were female (78.3%), middle-aged (40.4 years), and exceedingly obese (34.8%; Table 1 ). Most were otherwise healthy, with diabetes mellitus being the most common comorbidity (17.4%). There had been no prior significant surgical interventions other than drainage maneuvers, except for one patient in Romania where, before initiation of this study, the contralateral side had been closed with a circumflex scapular rotation flap. Extensive Hurley stage III disease was more often the impetus for this treatment in the United States (75.0%) than in Romania (45.5%), but both treated Hurley stage II disease, typically, if the patient desired a definitive and more rapid resolution of their suffering ( Table 2 ). Defect and flap dimensions were not routinely measured, as essentially the entire cupola of the axilla was removed with then a flap slightly exceeding the height of the defect and a width 50% greater.

All U.S. patients were discharged as outpatients, including the single patient with bilateral disease whose treatment required two separate admissions. Governmental protocols in Romania required that all patients be admitted postoperatively as inpatients, explaining why all three patients with bilateral HS had both axilla treated during the same admission. Re-admissions specifically for treatment of HS complications did not occur in any case in either country. Loss of even minor flap viability was not observed. However, complications were not uncommon ( Table 2 ), especially dehiscence of the lesser curvature closure of the flap (25.9%) where tension due to arm elevation was the greatest, whether the flap had an original transverse, oblique, or vertical orientation. This complication occurred more often in the United States (38.5%) than in Romania (14.3%). Local outpatient wound care proved to be sufficient to treat all complications. Nevertheless, recovery on average was prolonged up to 4 months for those individuals. No disease recurrences were noted for any patient during the overall mean follow-up period of 4 months. Physical therapy consultation was necessary to restore full shoulder range of motion only in a single individual in the United States, otherwise, this could be guided by the operating surgeon. Without exception, all patients were satisfied with the outcome regardless of any difficulties encountered, as the result was always superior to their preoperative status.

## Discussion

Surgery as a treatment modality for axillary HS should be a consideration only after all available nonoperative options have been explored, as recidivism can always occur. 12 Healing by secondary intention is a long-term process and requires intensive dressing changes that are painful. 8 Primary closure may be possible only if disease resection was not extensive enough. Skin grafts in this milieu may take unsuccessfully; but, if adequate, always risk contraction that would impair shoulder mobility. Flaps are technically more difficult but allow immediate closure of larger defects that will hasten healing, lessen discomfort, and promote earlier mobility. Many of the detriments of numerous other local flaps available for axillary resurfacing, 1 5 13 14 15 including the TDAP flap, which is considered the “workhorse” flap for HS in this region, 2 7 8 16 17 18 19 can be avoided using a keystone perforator island advancement flap. Although the TDAP flap, as does our axillary keystone flap, captures the same dorsal thoracic fascia territory and provides an excellent color and texture match for this region, the requisite perforator harvest must be meticulous and always tedious. 2 7 8 16 17 18 19 However minimized, total preservation of latissimus dorsi muscle function cannot occur. 2 7 Postoperative dressings and immobilization after a TDAP flap must always be maintained with caution for an indeterminate period for fear of crushing the very perforator required to maintain circulation and viability. 18 In comparison, a keystone flap can be executed relatively simply and quickly since no perforators ever need their specific identification or unveiling. 20 21 Since dependent on multiple perforators, it is virtually impossible to devascularize a keystone flap even after some subfascial elevation, thereby ensuring flap survival. 20 21

Some limitations in the use of a keystone perforator island advancement flap for resurfacing the axilla must be respected. Lack of local tissue extensibility in the asthenic individual precludes this choice for a large defect, but rarely is a problem with HS patients where the mobile adipose layer in obesity can actually be an advantage. A large keystone flap will result in significant scarring that cannot always be hidden with the arm adducted ( Fig. 1 ). Closure of the lesser curvature can be precarious, as dehiscence is the most common untoward event due to uncontrolled arm elevation, especially if the patient is not compliant.

### Conclusion

Consideration of multimodal therapy combining surgery with biological agents in the future may decrease further the degree of surgical intervention and risk of recurrence when treating HS. 4 Until then, the keystone perforator island advancement flap, as evidenced by this similar positive experience in both the United States and Romania, is a very simple, efficient, and reliable local flap option for the closure of the indeterminate but usually large axillary defect after radical excision of the ravages of HS.
